# The serum protein profile of early parity which induces protection against breast cancer

**DOI:** 10.18632/oncotarget.12757

**Published:** 2016-10-19

**Authors:** Christina Gutierrez Bracamontes, Rebecca Lopez-Valdez, Ramadevi Subramani, Arunkumar Arumugam, Sushmita Nandy, Venkatesh Rajamanickam, Vignesh Ravichandran, Rajkumar Lakshmanaswamy

**Affiliations:** ^1^ Center of Emphasis in Cancer Research, Department of Biomedical Sciences, Texas Tech University Health Sciences Center, Paul L. Foster School of Medicine, El Paso, TX 79905, USA; ^2^ Division of Genetic Epidemiology, University of Utah School of Medicine, Salt Lake City, UT 84112, USA; ^3^ Cancer Biology and Genetics Program, Memorial Sloan-Kettering Cancer Center, New York, NY 10065, USA; ^4^ Texas Tech University Health Sciences Center El Paso-Graduate School of Biomedical Sciences, El Paso, TX 79905, USA

**Keywords:** parity, breast cancer risk, serum proteins, protection, biomarkers

## Abstract

Early parity reduces the risk of breast cancer in women while nulliparity and late parity increase the risk of breast cancer. In order to translate this protection to women where early pregnancy is not feasible, much work has focused on understanding how parity confers protection against breast cancer, the molecular mechanisms by which this occurs is still not well understood. Healthy parous and nulliparous women were recruited for this study. We assessed serum protein profiles of early parous, late parous, and nulliparous women using the Phospho Explorer antibody array. Significantly altered proteins identified were validated by Western blot analysis. *In silico* analysis was performed with the data obtained. Our findings indicate increased phosphorylation levels of CDK1, AKT1 and Epo-R increased cell cycle and cell proliferation in late/nulliparous women. Increased levels of LIMK1, paxillin, caveolin-1, and tyrosine hydroxylase in late/nulliparous women demonstrate enhanced cell stress while decreased activity of p-p53 and pRAD51 in late/nulliparous women indicates decreased apoptosis and increased genomic instability. Further, increased levels of pFAK, pCD3zeta, pSTAT5B, MAP3K8 in early parous women favor enhanced innate/adaptive immunity. Overall, we have identified a unique protein signature that is responsible for the decreased risk of breast cancer and these proteins can also serve as biomarkers to predict the risk of breast cancer.

## INTRODUCTION

Breast cancer remains the most commonly diagnosed cancer in women worldwide [1, 2] and current therapies are plagued by the development of resistance and high toxicity [3]. Therefore, the best treatment approach would be to elucidate and employ strategies that effectively reduce the risk of breast cancer in women. This requires discovery of suitable biomarkers that can accurately predict a woman's risk of developing breast cancer and a woman's risk of disease progression if breast cancer is diagnosed. Pregnancy before the age of 25 reduces a woman's risk of developing breast cancer by as much as 50% [4]. On the contrary, a first pregnancy after the age of 35 is known to increase a woman's risk of developing breast cancer [5]. Despite concerted efforts to understand this phenomenon, the exact etiology behind parity-induced protection against breast cancer is not fully understood.

In previous epidemiologic studies and in studies using rodent animal models, we and others have shown that there are notable differences in the hormonal milieu of parous vs. nulliparous individuals and these differences account, in part, for parity-induced protection against breast cancer [6–13]. These prior studies identified alterations in pituitary-gonadal hormones as the primary contributors to the systemic effects of parity, which include differences in prolactin and growth hormone levels [6–11]. We and others have also shown that alterations in growth hormone signaling yields the most pronounced effect on breast cancer susceptibility in rodent models [9, 14–16]. In fact, growth hormone-deficient rats are completely resistant to mammary carcinogenesis [16]. Further, Abrams and colleagues demonstrated that when susceptible rat mammary epithelial cells were treated with carcinogen and transplanted into virgin or parous hosts, nearly all virgin host transplants developed tumors compared to a negligible number of parous host transplants [17]. Again, this demonstrates that parity-induced protection is largely accomplished by system-wide changes which impacts breast tissue in such a way as to render mammary epithelial cells refractory to carcinogenesis. Further, changes at the cellular level may also contribute to parity-induced protection [18, 19].

Given the strong evidence that protection against breast cancer in parous individuals is through systemic changes, it is surprising that virtually no recent study in human subjects has looked at parity-induced systemic changes. Most recent studies in human subjects and rodent animal models have focused on parity-induced changes at the tissue and cellular level [19–24]. Current studies in human subjects have compared 1) gene expression profiles of mammary tissue from parous and nulliparous women [12, 22]; and 2) gene expression profiles of mammary tissue from premenopausal women undergoing reduction mammoplasty and breast biopsy of benign breast lesions [21]. To our knowledge, the results presented here represent the largest study of completely normal healthy women that includes subjects stratified according to nulliparous, late parous and early parous status. The current study is also unique because we have assessed parity-induced systemic changes in the serum of normal healthy women not undergoing breast reduction surgeries, augmentations surgeries, or lesion biopsies. Here, we analyzed global changes in protein expression and identified serum protein profiles that are unique to 1) low-risk early parous women, and 2) high-risk nulliparous and late parous women. This study is critical for identification of breast cancer biomarkers that could effectively predict the risk and progression of breast cancer, as well as, offer important clues towards developing strategies to prevent breast cancer in highly susceptible women. We expect the unique protein signatures identified here will provide important insights for both the prevention and treatment of breast cancer in women.

## RESULTS

### Proteomic analysis

**Figure 1:**
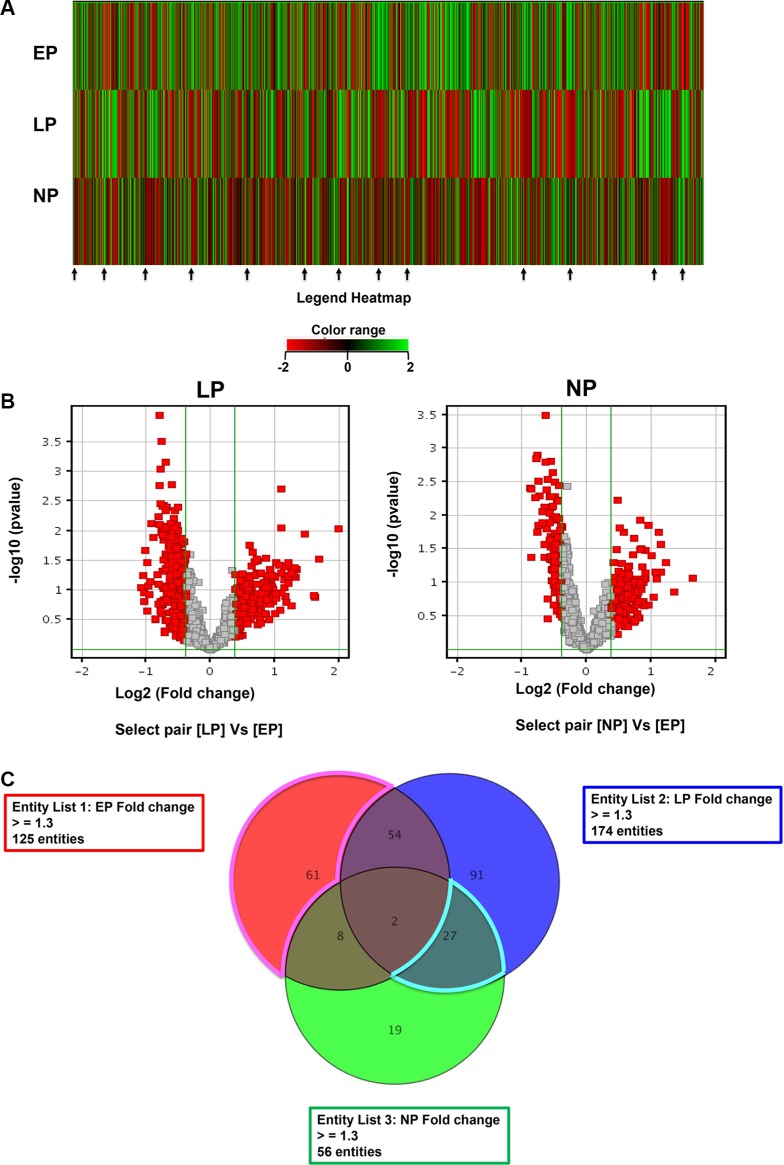
Analysis and comparison of the serum proteome of each group Using GeneSpring bioinformatics analysis software, (**A**) the average expression of each protein in each sample group was subjected to hierarchical clustering for early parous (EP), late parous (LP), and nulliparous (NP) subjects. The resulting heatmap indicates proteins that are differentially regulated in the serum of subjects from each group. Red indicates proteins that were downregulated at least two-fold and green indicates proteins that were upregulated at least two-fold. (**B**) Volcano plots of the data with a fold change cutoff of ≥ 1.3 and a *p*-value cutoff of ≤ 0.05 were generated. Highlighted in red are proteins that were either downregulated or upregulated in late parous or nulliparous samples with early parous samples serving as the control. (**C**) Using a fold change cutoff of ≥ 1.3 and a *p*-value cutoff of ≤ 0.05, a Venn diagram was constructed to find differentially regulated proteins of interest, namely those proteins differentially expressed only in EP samples (61 proteins) and those differentially coexpressed in LP and NP samples (27 proteins).

A total of 132 healthy volunteers—44 nulliparous subjects, 44 late parous subjects, and 44 early parous subjects—were recruited for this study [12]. Protein expression profiling was carried out using the Phospho-Explorer protein array. Proteomic analysis of the data obtained was performed using GeneSpring bioinformatics software and Metacore knowledge-based bioinformatics software. Raw protein expression values were first normalized and then subjected to a fold change cutoff of ± 1.3. Fold differences in protein expression with a *p*-value of ≤ 0.05 were considered significant. As expected, there were notable differences in the protein expression of each group (Figure 1A). Moreover, a large number of proteins were differentially regulated in late and nulliparous samples compared to early parous samples (Figure 1B) and a subset of these proteins were commonly expressed between late and nulliparous groups (Figure 1C). In order to distinguish proteins that may be involved in facilitating the protective effects of parity, we specifically focused on proteins with similar expression patterns among the two high risk groups (late and nulliparous) that were distinct from the expression pattern observed in early parous subjects. Of note, the majority of differentially expressed proteins appear to be downregulated in late/nulliparous subjects compared to early parous subjects. Many of these are highlighted by arrows in Figure 1A.

### Apoptosis and DNA damage responses

**Figure 2:**
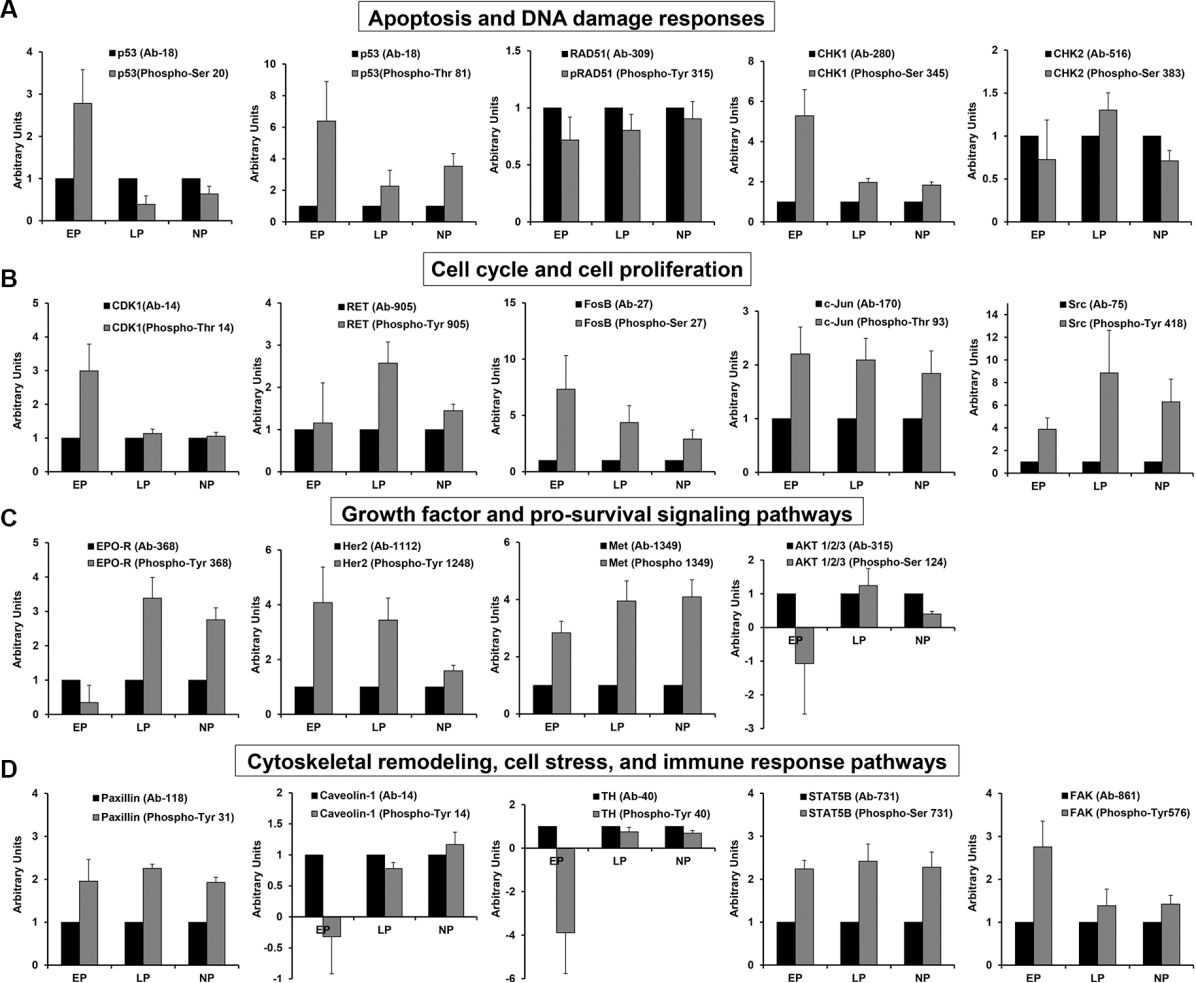
Comparison of active protein levels across groups Graphical representation of various proteins of interest which were differentially expressed in late/nulliparous individuals compared to early parous individuals. Each graph shows the ratio of total to phosphorylated protein for each group: early parous (EP); late parous (LP); nulliparous (NP). (**A**) Serum proteins involved in apoptosis and DNA damage responses. (**B**) Serum proteins that regulate various aspects of cell cycle progression and cell proliferation. (**C**) Serum proteins involved in the response to growth factors and involved in intracellular signaling pathways which enhance cell proliferation. (**D**) Serum proteins that regulate cell adhesion and migration, cell stress, and immune responses.

Several interesting patterns of protein expression immediately emerge from late/nulliparous vs. early parous serum protein profiles. First, expression levels of proteins critical for regulating apoptosis and repairing DNA damage appear to be decreased in the late/nulliparous groups in comparison to the early parous group. These include RAD51 and p53 (Table 1, Figure 2A). p53 is particularly important for cell cycle regulation and induction of apoptosis in response to DNA damage. When compared to early parous women, two phosphorylated forms of p53 critical for effective protein activity are downregulated in late and nulliparous women (Table 1, Figure 2A). Phosphorylation of p53 at serine 20 has been shown to alleviate MDM2-mediated inhibition of p53 [25] and this form of the protein is significantly downregulated in late/nulliparous women (Table 1). Similarly, p53 phosphorylated at threonine 81 stabilizes p53 in response to cell stress [26] and this form of the protein is also downregulated in late/nulliparous women (Table 1). This indicates that p53 is less stable and more prone to inhibition in late/nulliparous women. RAD51 phosphorylated at tyrosine 315 is also significantly downregulated in late/nulliparous women compared to early parous individuals (Table 1, Figure 2A), and this form of RAD51 has enhanced binding affinity for damaged DNA, which facilitates DNA repair [27]. This suggests that there may be an impaired DNA damage response in late/nulliparous women compared to early parous women.

**Table 1:** Serum proteins differentially expressed in late/nulliparous women compared to early parous women that are involved in apoptosis, cell cycle, DNA damage responses, and cell proliferation

Apoptosis				
Protein Name	Swiss Prot ID	Function	Fold Change LP vs EP	Fold Change NP vs EP
**Src (Ab-75)**	P12931	Gene transcription, immune responses, cell adhesion, cell cycle progression, apoptosis, migration, and transformation.	2.32	3.95
**CASP9 (Phospho-Thr125)**	P55211	Execution phase of apoptosis. Phosphorylation at this site inhibits apoptosis.	1.30	1.31
**14-3-3 zeta/beta (Ab-184/186)**	P63104/P31946	Cell cycle control, apoptosis, cellular signaling, stress responses, inflammation. Adapter protein that modulates protein function/stability/location.	−1.76	−1.57
**Calsenilin/KCNIP3 (Ab-63)**	Q9Y2W7	Calcium-binding protein, transcriptional repressor.	−7.53	−2.99
**ETK (Phospho-Tyr40)**	P51813	Signal transduction, actin reorganization, cell migration, cell proliferation and survival, cell adhesion, apoptosis	−1.56	−1.41
**MKP-1/2 (Phospho-Ser296)**	P28562/Q13115	Signal transduction, cell cycle control. Negative regulation of MAPK signaling. Phosphorylation at serine 296 induces MKP protein degradation.	−1.63	−1.38
**p53 (Phospho-Ser20)**	P04637	Cell cycle control, DNA damage response, apoptosis. Serine 20 phosphorylation may alleviate inhibition by MDM2.	−1.63	−1.49
**p53 (Phospho-Thr81)**	P04637	Cell cycle control, DNA damage response, apoptosis. Threonine 81 phosphorylation stabilizes p53 in stress responses.	−1.67	−1.60
**PAK1**	Q13153	Signal transduction, actin reorganization, cell adhesion/migration, apoptosis, immune responses.	−1.61	−1.48
**PAK1/2/3 (Ab-141)**	Q13153/Q13177/O75914	Signal transduction, actin reorganization, cell adhesion/migration, apoptosis, immune responses. PAK1/2 are necessary for efficient AKT translocation to cell membrane and AKT activation. PAK1/4 phosphorylate LIMK1 on threonine.	−1.48	−1.71
**TIE2 (Phospho-Tyr1108)**	Q02763	Angiogenesis, survival, proliferation, adhesion/migration/cell spreading. Cell surface receptor for angiopoietins. Ligand binding phosphorylates tyrosine 1108. Tyr1108 Important for DOK2 interactions and coupling downstream signaling.	−1.61	−1.49
**XIAP (Ab-87)**	P98170	Apoptosis inhibitor. Directly inhibits caspase activity. Major role in receptor-mediated apoptosis.	−1.54	−1.55
**Cell Cycle**				
**Calmodulin (Phospho-Thr79/Ser81)**	P62158	Calcium binding protein. Regulates certain kinases and phosphatases, regulates centrosome cycle/progression through cytokinesis. Phosphorylation inhibits activity.	4.58	4.75
**PKC theta (Ab-676)**	Q04759	Signal transduction, immune responses, cell cycle. T-cell activation and survival. NFkB and AP-1 activation.	2.41	2.17
**CaMK1-a (Ab-177)**	Q14012	Calcium triggered signaling. Regulates transcription factor activity, cell cycle, hormone production, cell differentiation, actin filament organization.	−1.52	−1.72
**CDK1/CDC2 (Phospho-Thr14)**	P24941	Cell cycle. Inhibition of cell cycle progression when phosphorylated at this site.	−1.84	−1.61
**Rb (Phospho-Ser795)**	P06400	Negative regulator of cell cycle. Phosphorylation inhibits activity.	−1.38	−1.55
**DNA Damage Responses**				
**CHK1 (Ab-280)**	Q14757	Cell cycle, DNA damage response. Critical for G2/M DNA damage checkpoints. Phosphorylates CDC25C to activate G2 arrest.	1.66	1.48
**CHK2 (Ab-516)**	Q9Z265	Cell cycle, DNA damage response. Stabilizes p-53 to activate G1 arrest. Phosphorylates CDC25C to activate G2 arrest. Also regulates CDC25A and CDC25B activity.	1.55	1.87
**RAD51 (Ab-309)**	Q06609	DNA repair, homologous recombination, DNA damage response. Tyrosine 315 phosphorylation enhances binding affinity towards damaged DNA.	−1.54	−1.31
**RAD51(Phospho-Tyr315)**	Q06609		−1.68	−1.45
**Cell Proliferation/Cell Growth**				
**AKT1 (Phospho-Ser124)**	P31749	Signal transduction, cell survival, cell growth, cell adhesion and motility, cytoskeletal remodeling, immunity, inflammation.	3.51	2.09
**ERK8 (Phospho-Thr175/Tyr177)**	Q8TD08	Cell proliferation and transformation, regulation of various nuclear receptors	5.67	5.10
**GSK3a-b (Ab-216/279)**	P49840/P49841	Regulation of energy metabolism, glycogen metabolism. Key roles in cell division, proliferation, motility, and survival. Phosphorylates tau and presenilin-1.	2.00	1.75
**IRS-1 (Ab-312)**	P35568	Insulin receptor signaling.	1.5	1.55
**IRS-1 (Phospho-Ser312)**	P35568	Signal transduction, cell survival/growth, cell adhesion/motility, cytoskeletal remodeling, immunity, inflammation.	1.39	1.30
**JAK-2 (Ab-221)**	O60674	Cytokine/growth factor signaling. Innate/adaptive immune signaling. Growth hormone, prolactin, leptin, insulin, and Epo-R signaling.	2.30	1.66
**RET (Ab-905)**	P07949	Signal transduction, cell growth, differentiation, transformation. Phosphorylates FAK1.	2.28	1.93
**Met (Phospho-Tyr1349)**	**P08581**	Hepatocyte growth factor receptor. Regulate EMT (epithelial-mesenchymal transition) Growth factor signaling, cell proliferation/scattering/morphogenesis/survival.	4.34	5.97
**Met (Phospho-Tyr1356)**	**P08581**		−1.3	−1.66
**EPO-R (Phospho-Tyr368)**	P19235	Dysregulation may affect the growth of certain tumors. Erythropoietin growth factor signaling. Activates JAK/STAT signaling.	1.84	1.83
**EPO-R (Ab-368)**	P19235		−1.58	−1.41
**c-Jun (Ab-170)**	P05412	Signal transduction, cell growth, stress responses, transformation. Dimerizes with Fos proteins to form the AP-1 transcription factor complex. Proto-oncogene that can transform cells both alone and with other cooperating oncogenes.	−1.64	−1.49
**c-Raf (Ab-296)**	P04049	Signal transduction, cell growth. Positively regulates cell growth in response to mitogens/growth factors. Proto-oncogene.	−2.01	−1.69
**DOK-1 (Phospho-Tyr362)**	Q99704	Signal transduction, protein docking. Adapter protein for multimolecular signaling complexes. Phosphorylated form negatively regulates insulin signaling.	−1.57	−1.70
**FosB (Ab-27)**	P53539	Cell proliferation, differentiation, transformation. Dimerizes with Jun family members to form the AP-1 transcription factor complex. Loss of FosB in breast cancer associated with hormone receptor negative status and high grading.	−6.28	−4.55
**FosB (Phospho-Ser27)**	P53539		−1.69	−1.44
**HER2 (Ab-1112)**	P04626	Growth factor signaling, protein synthesis enhancement, cell growth. No ligand binding ability but binds to other family members to stabilize receptor-ligand interaction and activate downstream signaling.	−1.67	−1.49
**HRS (Ab-334)**	O14964	Signal transduction, endosomal sorting, recycling and degradation of membrane receptors. Many growth factor receptors require interactions with HRS for inactivation. Absent or aberrant HRS function may underlie formation of various cancers due to constant growth factor receptor activation.	−1.63	−1.51
**JAK-1 (Ab-1022)**	P23458	Cytokine/growth factor signaling. IFN-alpha/beta/gamma singaling.	−1.40	−1.30
**MSK1 (Ab-581)**	O75582	Signal transduction, growth factor response, cell stress response. Dampens proinflammatory immune responses through IL-10 production.	−1.65	−1.52

EP, early parous. LP, late parous. NP, nulliparous.

In the case of p53, there appears to be a deficiency in p53 activation in late/nulliparous women based on the ratio of phosphorylated protein to total protein (Figure 2A). For RAD51, on the other hand, the ratio of total to phosphorylated protein remains the same for all three groups (Figure 2A), suggesting a deficiency in protein translation. Early parous individuals have much higher levels of active CHK1 (Figure 2A) while there is no significant difference in active CHK2 levels (Figure 2A).

### Cell cycle and cell proliferation

Another intriguing pattern of protein expression in late/nulliparous subjects is indicative of poorly controlled cell cycle progression which may lead to enhanced cell proliferation. This is reflected in the downregulation of CDK1 (p-Thr14) along with the upregulation of the RET and Src proto-oncogenes (Table 1, Figure 2B). Downregulation of HRS (hepatocyte growth factor-regulated tyrosine kinase substrate) may also play an important part in “poorly controlled” cell proliferation within these populations (Table 1). Phosphorylation of CDK1 at threonine 14 inhibits CDK1 activity thus inhibiting cell cycle progression [28]. Therefore, downregulation of this form of CDK1 suggests that cell cycle progression may actually be enhanced in late/nulliparous women. Further, despite significant variation within replicates which precludes statistical significance, there is a trend for increased phospho-RET and phospho-Src activation in late/nulliparous subjects (Figure 2B). In many cases, RET gain-of-function is associated with breast cancer formation through enhanced cell proliferation, migration and cell scattering, as well as, through enhanced cytokine production [29–32]. Likewise, Src hyperactivation fuels progression of various cancers [33] and plays a key role in breast cancer resistance to Herceptin therapy [34]. Therefore, overexpression of these proteins may render cells more susceptible to hyperactive signaling and eventually oncogenic transformation.

It is interesting that total protein levels of several proto-oncogenes were upregulated in early parous women, including c-Jun and FosB (Table 1). However, only in the case of FosB there is a corresponding trend for increased protein activity compared to late/nulliparous subjects (Figure 2B). Early parous serum protein profiles also indicate alteration of several growth factor signaling pathways such as the erythropoietin pathway (Epo-R), the human epidermal growth factor pathway (HER2, JAK1), and the hepatocyte growth factor pathway (Met) (Table 1). We actually see higher expression of active Epo-R and Met in late/nulliparous women and no difference in active Her2 between early and late parous individuals (Table 1, Figure 2C). This suggests that these pathways do not enhance cell growth above a normal basal level in early parous women compared to late/nulliparous women.

Further, there is strong evidence of better control over cell cycle progression and cell growth in early parous women based on the following findings: 1) upregulation of phosphorylated CDK1 (Thr14), 2) downregulation of phosphorylated AKT1 (Ser124), 3) downregulation of phosphorylated Epo-R (Tyr368), and 3) upregulation of HRS (Table 1, Figure 2B and 2C). Upregulation of the inhibitory form of CDK1 [28] coupled with downregulation of the form of AKT1 required for optimal downstream activation [35] likely reflects much tighter control over cell cycle progression in early parous individuals. Suppression of the active form of Epo-R also indicates more fine-tuned control of this signaling pathway in early parous women. Also, the HRS protein is more abundant in early parous subjects and is required by many growth factor receptors for appropriate receptor recycling, inactivation, and degradation [36]. This indicates that growth factor signaling is more well-balanced in early parous individuals.

### Cytoskeletal remodeling, cell stress and immune responses

A third serum protein expression profile that emerges from this data is the differential regulation of many proteins involved in cytoskeletal remodeling, cell adhesion and migration (Table 2). While a number of proteins in this category were downregulated, several key proteins were significantly upregulated in late/nulliparous subjects, including LIMK1, paxillin, and caveolin-1 (Table 2, Figure 2D). LIMK1, is important for stabilizing the actin cytoskeleton and thus in cell motility and cell cycle progression [37]. Likewise, paxillin is important in the regulation of focal adhesions/cell motility and serves as a docking protein for intracellular signaling [38]. LIMK1 overexpression has been noted in breast cancer progression [39], while paxillin overexpression has been shown to enhance breast cancer metastasis [40]. Caveolin-1 is a scaffolding protein found in certain types of lipid rafts that facilitate cellular signal transduction [41]. Caveolin-1 has been shown to be positively influence many biological processes including cell migration and cell stress responses [41, 42]. In fact, caveolin-1 is phosphorylated on tyrosine residues in response to cellular stress [42], and pTyr14-caveolin-1 is significantly upregulated in late/nulliparous women perhaps suggesting increased levels of cellular stress (Table 2, Figure 2D). Upregulation of tyrosine hydroxylase in late/nulliparous women further suggests enhanced cellular stress (Table 2, Figure 2D). Chronic social stress has been tied to increased tyrosine hydroxylase levels which catalyze catecholamine synthesis—a process frequently deregulated by chronic stress in both humans and animals [43].

**Table 2:** Serum proteins differentially expressed in late/nulliparous women compared to early parous women that are involved in cytoskeletal remodeling, cell stress, and immune responses

Cytoskeletal Remodeling				
Protein Name	Swiss Prot ID	Function	Fold Change LP vs EP	Fold Change NP vs EP
**Caveolin-1 (Phospho-Tyr14)**	Q03135	Cell migration, scaffolding protein in caveolae. Phosphorylated in focal adhesions.	2.24	2.25
**LIMK1 (Ab-508)**	P53667	Actin cytoskeleton stabilization, cell motility, cell cycle progression, differentiation. Activated by ROCK1, PAK1 and PAK4.	3.51	2.28
**Paxillin (Ab-118)**	P49023	Cytoskeletal remodeling, cell spreading/motility, docking protein for signal transduction. Direct interactions with PAK3 and indirect interactions with all other PAK family members.	2.55	1.86
**CaMK1-a (Ab-177)**	Q14012	Calcium triggered signaling. Regulates transcription factor activity, cell cycle, hormone production, cell differentiation, actin filament organization.	−1.52	−1.72
**ETK (Phospho-Tyr40)**	P51813	Signal transduction, actin reorganization, cell migration, cell proliferation and survival, cell adhesion, apoptosis	−1.56	−1.41
**FAK (Phospho-Tyr576)**	Q05397	Cytoskeletal remodeling, cell adhesion, chemotaxis, ECM signal transduction, cell cycle, development. Phosphorylated upon activation. Tyrosine 576 is phosphorylated by Src. Critical role in Src-induced EMT.	−1.57	−1.42
**PAK1/2/3 (Ab-141)**	Q13153/Q13177/O75914	Signal transduction, actin reorganization, cell adhesion/migration, apoptosis, immune responses. PAK1/2 are necessary for efficient AKT translocation to cell membrane and AKT activation. PAK1/4 phosphorylate LIMK1 on threonine.	−1.48	−1.71
**PAK3 (Phospho-Ser154)**	O75914	Signal transduction, cell morphology, cell migration.	−1.44	−1.86
**PKD1/PKCmu (Ab-910)**	P98161	Serine/threonine kinase that enhances cell adhesion in various cell-types. Prevents epithelial to mesenchymal transition in mammary epithelial cells.	−2.71	−3.70
**PYK2 (Ab-881)**	Q14289	ECM signal transduction, chemotaxis. Controls disassembly of VE-cadherin cell-cell junctions.	−1.51	−1.85
**TIE2 (Phospho-Tyr1108)**	Q02763	Angiogenesis, cell survival, proliferation, migration, adhesion, and cell spreading. Cell surface receptor for angiopoietins. Ligand binding induces tyrosine 1108 phosphorylation which is important for DOK2 interactions and coupling of downstream signal transduction.	−1.61	−1.49
**VAV1 (Ab-160)**	P15498	Signal transduction, actin remodeling, immunity. Guanine nucleotide exchange factor for Rho family GTPases.	−1.71	−1.63
**Cell Stress**				
**Tyrosine Hydroxylase (Phospho-Ser40)**	P07101	Catecholamine biosynthesis, stress responses. Phosphorylation at this site results in the most potent induction of TH activity.	2.84	2.68
**14-3-3 zeta/beta (Ab-184/186)**	P63104/P31946	Cell cycle control, apoptosis, cellular signaling, stress responses, inflammation. Adapter protein that modulates protein function/stability/location.	−1.76	−1.57
**MKK7/MAP2K7 (Phospho-Thr275)**	O14733	Signal transduction, inflammation, apoptosis, stress responses. Activates JNK signaling.	−1.63	−1.46
**MAP3K7/TAK1 (Ab-439)**	O43318	Signal transduction, transcription regulation, apoptosis, stress responses. Important in response to TGF beta and BMP signaling.	−1.48	−1.53
**Immune Responses**				
**PKC theta (Ab-676)**	Q04759	Signal transduction, immune responses, cell cycle. T-cell activation and survival. NFkB and AP-1 activation.	2.41	2.17
**CD3Z (Phospho-Tyr142)**	P20963	Immunity (TCR signaling), antigen recognition. Tyrosine phosphorylation occurs after T cell receptor triggering.	−1.60	−1.51
**MEF2C (Phospho-Ser396)**	Q06413	Immune responses, apoptosis, development. Transcription factor/activator. Phosphorylation at serine 396 inhibits transcriptional activity.	−1.63	−1.38
**MAP3K8/COT (Ab-400)**	P41279	Signal transduction, immune responses (TCR signaling), transformation. Proto-oncogene that activates MAPK, JNK, and NFkB signaling.	−1.46	−1.52
**Opioid Receptor (Ab-375)**	P35372	Respiration, cardiovascular functions, feeding, learning and memory, hormone secretion and immune functions.	−3.19	−1.62
**STAT5B (Phospho-Ser731)**	P51692	TCR signaling, T cell responses, cytokine signaling, growth hormone signaling. Serine phosphorylation occurs in lymphocytes in response to IL-2 stimulation.	−1.97	−1.31

EP, early parous. LP, late parous. NP, nulliparous.

Compared to late/nulliparous women, early parous subjects demonstrate upregulation of FAK/PAK and MAPK signaling (Table 2). Further, various proteins important for T-cell and NK cell mediated immunity are expressed at higher levels in early parous serum samples, including: CD3 zeta (T cell receptor signaling [44]); STAT5B (T and NK cell immunity [45]); and MAP3K8 (T cell IFN-gamma production [46]) (Table 2, Figure 2D).

### Validation of protein array results

**Figure 3:**
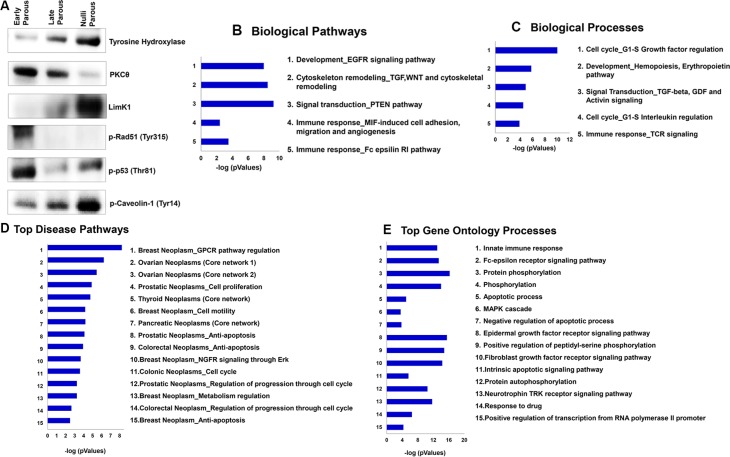
Confirmation of proteomic changes using Western blot and MetaCore knowledge-based bioinformatics analysis of the late/nulliparous serum proteome (**A**) Western blot analysis of total Tyrosine Hydroxylase, PKCθ, LimK1 and phosphorylated p-Rad51 (Tyr315), p-p53 (Thr81), and p-Caveolin1 (Tyr 14) in early parous (EP), late parous (LP), and nulliparous (NP) serum samples show a similar trend as that expressed in the protein array. (**B** and **C**) Top five biological pathways and top five biological processes in late and nulliparous women compared to early parous women. Functional and gene ontology enrichment analysis and network analysis was carried out to assess biological functions associated with protein profiles that likely play a role in parity-induced protection against breast cancer. (**D**) Top fifteen disease pathways according to known biomarkers in LP and NP women compared to EP women. (**E**) Top fifteen GO (gene ontology) processes in LP and NP women compared to Bar length is a reflection of significance and represents the negative logarithm of the enrichment *p*-value determined by MetaCore pathway enrichment analysis.

The final validation of our protein array results was accomplished through Western blot analysis of a subset of proteins chosen based on reproducibility in initial analyses, statistical significance, and biological relevance to parity-induced protection. These proteins included tyrosine hydroxylase, PKC theta, LimK1, pRad51 (Tyr315), p-p53 (Thr81), and pCaveolin-1 (Tyr 14) (Figure 3A). As demonstrated in the representative Western blots, the pattern of protein expression in serum samples assayed via Western blot closely mirrors the expression pattern observed in our protein array results. Although there was biological variation between individual replicates, the overall expression pattern remained similar. This confirms the integrity of the protein array data and indicates the accuracy and reproducibility of our proteomic analysis.

**Figure 4:**
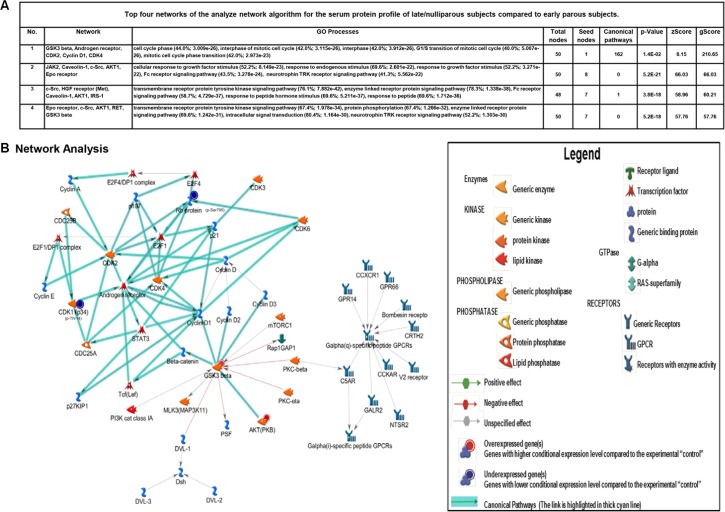
Bioinformatics analysis of altered networks in late/nulliparous serum proteome (**A** and **B**) Top four networks of the analyze network algorithm for the serum protein profile of late/nulliparous subjects compared to early parous subjects. Top scoring network of the analyze network algorithm of late and nulliparous serum protein profiles. Directional edges are marked with arrows that are either green (activation) or red (inhibition). Arrows highlighted in teal blue define the presence of canonical pathways with well-established signaling pathways.

Innate and adaptive immunity represent many of the top scoring gene ontology and biological pathways more significantly associated with proteins expressed at higher levels in early parous individuals. Further, early parous protein expression patterns demonstrate enrichment for gene ontology/biological processes that regulate cell cycle progression, apoptosis, protein phosphorylation, and transcription (Figure 3B–3E). Late/nulliparous protein expression patterns, on the other hand, were primarily associated with growth factor signaling and hormone and metabolic signaling (Figure 4A and 4B). All together, these results indicate that early parous populations may benefit from a greater level of “biological control” over basic cellular pathways governing everything from cell proliferation to immunity.

## DISCUSSION

The objective of this study was to survey differences in protein expression in the serum of normal healthy women to determine key differences in protein expression patterns in early parous vs. late and nulliparous women. This is critical, because examining differential protein expression according to this stratification allows us to distinguish parity-induced changes that increase or decrease breast cancer risk from changes that may be parity-associated but do not impact breast cancer risk. Changes in protein expression patterns that are unique to early parous women compared to those commonly shared by late parous and nulliparous women are likely related to reduce the risk of breast cancer.

Indeed, here we identified specific patterns of systemic protein expression unique to high-risk late and nulliparous women compared to low-risk early parous women. We expect that this distinct pattern reduces the risk of breast cancer, and also allows us to identify biomarkers useful in predicting breast cancer risk. The molecular signatures identified here will also help in the design of safe and non-invasive breast cancer prevention strategies for those individuals where early parity is not feasible.

The patterns of protein expression unique to late/nulliparous individuals revealed a protein signature associated with enhanced cell proliferation, enhanced cellular stress, and impaired DNA repair mechanisms. Metacore knowledge-based bioinformatics analysis further revealed that this protein expression signature is strongly associated with the development of various cancers including many forms of breast cancer. In fact, the top scoring network for this protein signature reveals a potential contribution from androgen receptor signaling and various G-protein coupled receptor signaling pathways. This is interesting because androgens are necessary precursors for estrogen synthesis and androgen receptor signaling itself has been demonstrated to increase cell proliferation and breast cancer progression [47]. This suggests an overall higher level of steroid hormone signaling in late/nulliparous women compared to early parous women. Earlier Asztalos et al. [21] reported that estrogen and progesterone signaling was downregulated in the parous breast compared to the nulliparous breast. Therefore, parity not only results in local suppression of steroid hormone signaling in breast tissue [21], but according to our results, also suppresses steroid hormone signaling systemically throughout the body. Another very interesting observation from this study is the apparent enhancement of immune defense regulation in early parous women compared to late/nulliparous women (Figure 3B and 3C). Also intriguing is the enhanced level of transcriptional and posttranscriptional regulation observed in early parous individuals—an observation which coincides with the findings of Peri et al. in breast tissue mRNA profiles [22]. This demonstrates that a tighter level of transcriptional regulation is observed at both the local tissue level [22] and systemically in early parous women. Therefore, this likely represents another important protective effect of early parity. But, what may also be of paramount importance to parity-related protection against breast cancer is the increased level of immune system regulation observed in early parous women. Our study reveals a significant contribution from both innate and adaptive immunity in the early parous proteome. We believe this unique signature may actually inhibit breast cancer in early parous women through increased immune surveillance, which would more effectively eliminate any breast epithelial cells that may undergo malignant transformation. Indeed, various epidemiologic studies demonstrate that heightened immunity is associated with protection from various cancers [48, 49]. It is therefore entirely possible that parity may have a similar effect on immunity and this heightened immunity may be a key aspect of parity-mediated protection against breast cancer.

In conclusion, this study demonstrates that late/nulliparous individuals appear to have higher baseline levels of cell proliferation and possibly cellular stress. In addition, there is also decreased cell cycle/apoptosis regulation and impaired DNA damage responses. These characteristics ultimately results in “non-specific” and “uncontrolled” cell proliferation with a much greater potential for malignant transformation in late and nulliparous women. In contrast, early parous individuals benefit from enhanced apoptosis/cell cycle regulation, DNA repair mechanisms, and immune defenses. We hypothesize that this translates into much more “well-controlled” cell proliferation where any malignantly transformed cells are more efficiently eliminated through several mechanisms including appropriate DNA repair and recovery or through programmed cell death and immune detection/elimination. This study, together with other recent reports [21, 22], provides a strong foundation for precisely defining the protein expression signature associated with decreased breast cancer in early parous women. We expect that this will shortly result in the identification of biomarkers suitable for predicting breast cancer risk and progression and will also lead to the development of effective breast cancer prevention and treatment strategies.

## MATERIALS AND METHODS

### Ethics statement

All experiments performed were reviewed and approved by the Texas Tech University Health Sciences Center Institutional Review Board and Institutional Biosafety Committee.

### Antibodies and reagents

Rabbit polyclonal anti-caveolin 1 (phosphor Y14) (ab38468), rabbit polyclonal anti-LimK1 (ab95186), rabbit monoclonal anti-PKC theta (ab110728) ([EPR1487(2)], rabbit polyclonal anti-Aurora A (ab1287), rabbit monoclonal anti-S6K (E175) (ab32359), and rabbit monoclonal anti-Mannose 6 phosphate receptor (cation independent) (ab124767) were purchased from Abcam Inc (Cambridge, MA, USA). Phospho Explorer antibody array and array assay kits were purchased from Full Moon Biosystems (Sunnyvale, CA, USA). A magnetic serum albumin depletion kit (cat no. LSKMAGL05) was purchased from EMD Millipore (Billerica, MA, USA). Pre-cast gels were purchased from Bio-Rad Inc. (Hercules, CA, USA).

### Volunteer criteria and samples

Healthy parous and nulliparous women between the ages of 40 and 60 yr. were recruited for this study at the Dr. Susan Love Research foundation in Santa Monica, CA [12]. Protocols for obtaining patient consent to donate venous blood and for the preservation of patient confidentiality, no patient identities were obtained. This protocol was reviewed and approved by the Institutional Review Board of Texas Tech University Health Science Center in El Paso, TX and by the Institutional Review Board of the Dr. Susan Love Foundation. Blood samples were drawn, processed to extract serum, the resulting serum samples were aliquoted and preserved at −80°C until further processing. Recruited women were divided into the following groups: early parous (first full-term pregnancy ≤ 25 yr.), late parous (first full-term pregnancy ≥ 35 yr.) and nulliparous (never undergone a full-term pregnancy). For parous women, samples were collected at least 5 years after the last full-term pregnancy. Only women who had a history of regular menses and who had not regularly taken medications or oral contraceptives in the six months prior to sample collection were admitted to the study. Subjects with liver disease, renal disease, or endocrine problems were excluded from the study.

### Phospho Explorer antibody array

Phospho Explorer antibody arrays were used to assess differences in serum protein profiles between the three groups. Protein array assays were performed according to the manufacturer's instructions. Briefly, whole serum was depleted of albumin (see below), diluted in labeling buffer (1:15), and mixed with Biotin/DMF labeling solution. Biotinylated proteins from each sample were then incubated on individual antibody-coated slides, followed by fluorescence labeling with Cy3-streptavidin (GE Healthcare). The slides were scanned on an Axon GenePix Array Scanner (Molecular Devices) to detect bound proteins based on fluorescence intensity. Fluorescence intensity of each protein array was normalized and analyzed using GeneSpring software v. 12.5 (Agilent, Santa Clara, CA, USA).

### Serum albumin depletion

Upon collection, blood samples were centrifuged in serum separator tubes and serum samples were stored at −80°C until further analysis. In order to minimize interference from the most abundant serum proteins, serum albumin was removed using the PureProteome albumin magnetic beads kit. Briefly, beads were washed twice with 1X PBS; human serum was diluted (1:15) in 1X PBS and added to the magnetic beads. The mixture was then incubated for an hour at room temperature on a rotator and beads were separated from the albumin-depleted serum according to the manufacturer's instructions.

### Western blot analysis

For Western blot, 24 microliters of albumin-depleted serum was separated on 4–20% sodium dodecyl sulfate polyacrylamide gels and transferred onto PVDF membranes. The membranes were then probed with select primary antibodies (as per manufacturer's instructions) overnight at 4°C. Secondary monoclonal anti-rabbit antibodies conjugated to horseradish peroxidase (Sigma Aldrich Inc, St Louis, MO, USA) were used to detect the proteins of interest with SuperSignal West Femto Maximum Sensitivity Substrates (Thermo Fisher Scientific Co, Hanover Park, IL, USA). Chemiluminescent signals were visualized digitally on the ImageQuant LAS 4000 digital imaging system (GE Healthcare BioSciences Corp, Piscataway, NJ, USA).

### Statistical analysis

Differences in the expression of individual proteins were compared between groups using an un-paired Student's *t*-test. Volcano plots and Venn diagrams were generated using a fold change cutoff of ≥ 1.3. Asymptotic *p*-value computation and multiple correction testing were done using the Benjamini-Hochberg procedure. Only the proteins that had a 30% of higher difference in expression and also had a value of *p* < 0.05 was considered as statistically significant.

## References

[1] DeSantis C, Ma J, Bryan L, Jemal A (2014). Breast cancer statistics, 2013. CA Cancer J Clin.

[2] Soerjomataram I, Lortet-Tieulent J, Parkin DM, Ferlay J, Mathers C, Forman D, Bray F (2012). Global burden of cancer in 2008: a systematic analysis of disability-adjusted life-years in 12 world regions. Lancet.

[3] de la Mare JA, Contu L, Hunter MC, Moyo B, Sterrenberg JN, Dhanani KC, Mutsvunguma LZ, Edkins AL (2014). Breast cancer: current developments in molecular approaches to diagnosis and treatment. Recent Pat Anticancer Drug Discov.

[4] Henderson BE, Powell D, Rosario I, Keys C, Hanisch R, Young M, Casagrande J, Gerkins V, Pike MC (1974). An epidemiologic study of breast cancer. J Natl Cancer Inst.

[5] Trichopoulos D, Hsieh CC, MacMahon B, Lin TM, Lowe CR, Mirra AP, Ravnihar B, Salber EJ, Valaoras VG, Yuasa S (1983). Age at any birth and breast cancer risk. International journal of cancer. Int J Cancer.

[6] Bernstein L, Ross RK, Pike MC, Brown JB, Henderson BE (1990). Hormone levels in older women: a study of post-menopausal breast cancer patients and healthy population controls. Br J Cancer.

[7] Pike MC, Gerkins VR, Casagrande JT, Gray GE, Brown J, Henderson BE (1979). The hormonal basis of breast cancer. Cancer Inst Monogr.

[8] Swanson SM, Guzman RC, Collins G, Tafoya P, Thordarson G, Talamantes F, Nandi S (1995). Refractoriness to mammary carcinogenesis in the parous mouse is reversible by hormonal stimulation induced by pituitary isografts. Cancer Lett.

[9] Thordarson G, Jin E, Guzman RC, Swanson SM, Nandi S, Talamantes F (1995). Refractoriness to mammary tumorigenesis in parous rats: is it caused by persistent changes in the hormonal environment or permanent biochemical alterations in the mammary epithelia?. Carcinogenesis.

[10] Bernstein L, Pike MC, Ross RK, Judd HL, Brown JB, Henderson BE (1985). Estrogen and sex hormone-binding globulin levels in nulliparous and parous women. J Natl Cancer.

[11] Yu MC, Gerkins VR, Henderson BE, Brown JB, Pike MC (1981). Elevated levels of prolactin in nulliparous women. Br J Cancer.

[12] Gutierrez CM, Lopez-Valdez R, Subramani R, Arumugam A, Nandy S, Rajamanickam V, Ravichandran V, Lakshmanaswamy R (2015). A Breast Tissue Protein Expression Profile Contributing to Early Parity-Induced Protection Against Breast Cancer. Cell Physiol Biochem.

[13] Arumugam A, Subramani R, Nandy S, Lopez R, Boopalan T, Lakshmanaswamy R (2014). Parity and short-term estradiol treatment utilizes similar cellular mechanisms to confer protection against breast cancer. Cell Physiol Biochem.

[14] Thordarson G, Semaan S, Low C, Ochoa D, Leong H, Rajkumar L, Guzman RC, Nandi S, Talamantes F (2004). Mammary tumorigenesis in growth hormone deficient spontaneous dwarf rats; effects of hormonal treatments. Breast Cancer Res Treat.

[15] Thordarson G, Slusher N, Leong H, Ochoa D, Rajkumar L, Guzman R, Nandi S, Talamantes F (2004). Insulin-like growth factor (IGF)-I obliterates the pregnancy-associated protection against mammary carcinogenesis in rats: evidence that IGF-I enhances cancer progression through estrogen receptor-alpha activation via the mitogen-activated protein kinase pathway. Breast Cancer Res.

[16] Swanson SM, Unterman TG (2002). The growth hormone-deficient Spontaneous Dwarf rat is resistant to chemically induced mammary carcinogenesis. Carcinogenesis.

[17] Abrams TJ, Guzman RC, Swanson SM, Thordarson G, Talamantes F, Nandi S (1998). Changes in the parous rat mammary gland environment are involved in parity-associated protection against mammary carcinogenesis. Anticancer Res.

[18] D'Cruz CM, Moody SE, Master SR, Hartman JL, Keiper EA, Imielinski MB, Cox JD, Wang JY, Ha SI, Keister BA, Chodosh LA (2002). Persistent parity-induced changes in growth factors, TGF-beta3, and differentiation in the rodent mammary gland. Mol Endocrinol.

[19] Verlinden I, Gungor N, Wouters K, Janssens J, Raus J, Michiels L (2005). Parity-induced changes in global gene expression in the human mammary gland. Eur J Cancer Prev.

[20] Uehara N, Unami A, Kiyozuka Y, Shikata N, Oishi Y, Tsubura A (2006). Parous mammary glands exhibit distinct alterations in gene expression and proliferation responsiveness to carcinogenic stimuli in Lewis rats. Oncol Rep.

[21] Asztalos S, Gann PH, Hayes MK, Nonn L, Beam CA, Dai Y, Wiley EL, Tonetti DA (2010). Gene expression patterns in the human breast after pregnancy. Cancer Prev. Res. (Phila).

[22] Peri S, de Cicco RL, Santucci-Pereira J, Slifker M, Ross EA, Russo IH, Russo PA, Arslan AA, Belitskaya-Levy I, Zeleniuch-Jacquotte A, Bordas P, Lenner P, Ahman J (2012). Defining the genomic signature of the parous breast. BMC Med Genomics.

[23] Russo J, Santucci-Pereira J, de Cicco RL, Sheriff F, Russo PA, Peri S, Slifker M, Ross E, Mello ML, Vidal BC, Belitskaya-Levy I, Arslan A, Zeleniuch-Jacquotte A (2012). Pregnancy-induced chromatin remodeling in the breast of postmenopausal women. Int J Cancer.

[24] Blakely CM, Stoddard AJ, Belka GK, Dugan KD, Notarfrancesco KL, Moody SE, D'Cruz CM, Chodosh LA (2006). Hormone-induced protection against mammary tumorigenesis is conserved in multiple rat strains and identifies a core gene expression signature induced by pregnancy. Cancer Res.

[25] Brooks CL, Gu W (2006). p53 ubiquitination: Mdm2 and beyond. Mol Cell.

[26] Buschmann T, Potapova O, Bar-Shira A, Ivanov VN, Fuchs SY, Henderson S, Fried VA, Minamoto T, Alarcon-Vargas D, Pincus MR, Gaarde WA, Holbrook NJ, Shiloh Y (2001). Jun NH2-terminal kinase phosphorylation of p53 on Thr-81 is important for p53 stabilization and transcriptional activities in response to stress. Mol Cell Biol.

[27] Shimizu H, Popova M, Fleury F, Kobayashi M, Hayashi N, Sakane I, Kurumizaka H, Venkitaraman AR, Takahashi M, Yamamoto K (2009). c-ABL tyrosine kinase stabilizes RAD51 chromatin association. Biochem Biophys Res Commun.

[28] Chow JP, Poon RY, Ma HT (2011). Inhibitory phosphorylation of cyclin-dependent kinase 1 as a compensatory mechanism for mitosis exit. Mol Cell Biol.

[29] Gattelli A, Nalvarte I, Boulay A, Roloff TC, Schreiber M, Carragher N, Macleod KK, Schlederer M, Lienhard S, Kenner L, Torres-Arzayus MI, Hynes NE (2013). Ret inhibition decreases growth and metastatic potential of estrogen receptor positive breast cancer cells. EMBO Mol Med.

[30] Morandi A, Martin LA, Gao Q, Pancholi S, Mackay A, Robertson D, Zvelebil M, Dowsett M, Plaza-Menacho I, Isacke CM (2013). GDNF-RET signaling in ER-positive breast cancers is a key determinant of response and resistance to aromatase inhibitors. Cancer Res.

[31] Spanheimer PM, Cyr AR, Gillum MP, Woodfield GW, Askeland RW, Weigel RJ (2014). Distinct pathways regulated by RET and estrogen receptor in luminal breast cancer demonstrate the biological basis for combination therapy. Ann Surg.

[32] Esseghir S, Todd SK, Hunt T, Poulsom R, Plaza-Menacho I, Reis-Filho JS, Isacke CM (2007). A role for glial cell derived neurotrophic factor induced expression by inflammatory cytokines and RET/GFR alpha 1 receptor up-regulation in breast cancer. Cancer Res.

[33] Irby RB, Yeatman TJ (2000). Role of Src expression and activation in human cancer. Oncogene.

[34] Zhang S, Huang WC, Li P, Guo H, Poh SB, Brady SW, Xiong Y, Tseng LM, Li SH, Ding Z, Sahin AA, Esteva FJ, Hortobagyi GN (2011). Combating trastuzumab resistance by targeting SRC, a common node downstream of multiple resistance pathways. Nat Med.

[35] Bellacosa A, Chan TO, Ahmed NN, Datta K, Malstrom S, Stokoe D, McCormick F, Feng J, Tsichlis P (1998). Akt activation by growth factors is a multiple-step process: the role of the PH domain. Oncogene.

[36] Lloyd TE, Atkinson R, Wu MN, Zhou Y, Pennetta G, Bellen HJ (2002). Hrs regulates endosome membrane invagination and tyrosine kinase receptor signaling in Drosophila. Cell.

[37] Manetti F (2012). LIM kinases are attractive targets with many macromolecular partners and only a few small molecule regulators. Med Res Rev.

[38] Zaidel-Bar R, Milo R, Kam Z, Geiger B (2007). A paxillin tyrosine phosphorylation switch regulates the assembly and form of cell-matrix adhesions. J Cell Sci.

[39] McConnell BV, Koto K, Gutierrez-Hartmann A (2011). Nuclear and cytoplasmic LIMK1 enhances human breast cancer progression. Mol Cancer.

[40] Deakin NO, Turner CE (2011). Distinct roles for paxillin and Hic-5 in regulating breast cancer cell morphology, invasion, and metastasis. Mol Biol Cell.

[41] Quest AF, Lobos-Gonzalez L, Nunez S, Sanhueza C, Fernandez JG, Aguirre A, Rodriguez D, Leyton L, Torres V (2013). The caveolin-1 connection to cell death and survival. Curr Mol Med.

[42] Volonte D, Galbiati F, Pestell RG, Lisanti MP (2001). Cellular stress induces the tyrosine phosphorylation of caveolin-1 (Tyr(14)) via activation of p38 mitogen-activated protein kinase and c-Src kinase. Evidence for caveolae, the actin cytoskeleton, and focal adhesions as mechanical sensors of osmotic stress. J Biol Chem.

[43] Kvetnansky R, Lu X, Ziegler MG (2013). Stress-triggered changes in peripheral catecholaminergic systems. Adv Pharmacol.

[44] Clark JM, Annenkov AE, Panesar M, Isomaki P, Chernajovsky Y, Cope AP (2004). T cell receptor zeta reconstitution fails to restore responses of T cells rendered hyporesponsive by tumor necrosis factor alpha. Natl Acad Sci USA.

[45] Lin JX, Li P, Liu D, Jin HT, He J, Ata Ur Rasheed M, Rochman Y, Wang L, Cui K, Liu C, Kelsall BL, Ahmed R, Leonard WJ (2012). Critical Role of STAT5 transcription factor tetramerization for cytokine responses and normal immune function. Immunity.

[46] Watford WT, Hissong BD, Durant LR, Yamane H, Muul LM, Kanno Y, Tato CM, Ramos HL, Berger AE, Mielke L, Pesu M, Solomon B, Frucht DM (2008). Tpl2 kinase regulates T cell interferon-gamma production and host resistance to Toxoplasma gondii. J Exp Med.

[47] Chang C, Lee SO, Yeh S, Chang TM (2014). Androgen receptor (AR) differential roles in hormone-related tumors including prostate, bladder, kidney, lung, breast and liver. Oncogene.

[48] Senchina DS, Kohut ML (2007). Immunological outcomes of exercise in older adults. Clin Interv Aging.

[49] Batty D, Thune I (2000). Does physical activity prevent cancer? Evidence suggests protection against colon cancer and probably breast cancer. BMJ.

